# Daily Associations Between Perceived Stress, Pain Intensity, and Alcohol Use Among Veterans With and Without Moderate‐to‐Severe Chronic Pain

**DOI:** 10.1002/smi.70153

**Published:** 2026-02-22

**Authors:** Shaddy K. Saba, John J. Prindle, Aysha Allahverdiyeva, Daniel Leightley, Eric R. Pedersen, Carl A. Castro, Jordan P. Davis

**Affiliations:** ^1^ New York University Silver School of Social Work New York New York USA; ^2^ University of Southern California Suzanne Dworak‐Peck School of Social Work Los Angeles California USA; ^3^ New York University Center for Data Science New York New York USA; ^4^ Department of Population Health Sciences King's College London School of Life Course and Population Sciences London UK; ^5^ University of Southern California Keck School of Medicine Los Angeles California USA; ^6^ RAND Santa Monica California USA

**Keywords:** mental health, nociception/pain perception, substance use

## Abstract

Chronic pain is prevalent among military veterans and commonly presents with perceived stress and alcohol use. Allostatic load models suggest moderate‐severe chronic pain may reflect a state of physiological dysregulation with heightened associations between pain and behavioural health symptoms. Yet little is known about daily associations between pain intensity, perceived stress, and alcohol use in veterans with and without moderate‐severe chronic pain. This study examined day‐to‐day associations between pain intensity, perceived stress, and alcohol use among veterans, and whether associations differ between those with moderate‐severe chronic pain and those with less severe pain. A sample of United States military veterans (*n* = 74) completed smartphone‐based daily diary surveys for up to 3 months, providing 4307 days of data. Multi‐group dynamic structural equation modelling examined within‐person, day‐to‐day associations between symptoms, among veterans with moderate‐severe chronic pain and those with less severe pain. Among veterans with moderate‐severe chronic pain, bidirectional positive day‐to‐day associations emerged between pain intensity and perceived stress (*b* = 0.06–0.13), and between perceived stress and alcohol use (*b* = 0.05–0.07). Pain intensity also predicted increased next‐day alcohol use (*b* = 0.04). Perceived stress appeared to act as a mechanism linking alcohol use to subsequent pain intensity (*b* = 0.01). For veterans with less severe pain, symptom associations differed markedly; higher perceived stress predicted lower next‐day pain intensity (*b* = −0.05). Among veterans with moderate‐severe chronic pain, day‐to‐day associations between pain intensity, perceived stress, and alcohol use appear more entangled. These veterans may experience heightened stress reactivity and poorer coping, requiring tailored interventions to monitor and address day‐to‐day symptom fluctuations.

## 1 Introduction

Military veterans experience high rates of chronic pain (physical pain lasting more than 3 months (Treede et al. 2015)), which is often associated with multi‐morbid behavioural health challenges such as substance use problems and stress‐related conditions (Goulet et al. 2016; Qureshi et al. 2023; Maleki et al. 2019; Otis et al. 2010). Over 30% of United States (US) veterans have chronic pain (Goulet et al. 2016; Qureshi et al. 2023), partly due to physically demanding military experiences including training and combat injuries and carrying heavy loads (Stratton et al. 2014; Fox B et al. 2020). Contemporary biopsychosocial models propose that chronic pain is influenced not only by these physical experiences but also by behavioural health factors (Gatchel et al. 2007), with perceived stress and substance use being two behavioural health factors that have demonstrated marked associations with pain (Saba et al. 2024; De Aquino et al. 2024). Prior work suggests that perceived stress—the belief that one's situation is unpredictable or beyond their ability to cope—can worsen pain (Saba et al. 2024). Some veterans report using substances such as alcohol to relieve pain (Vowles et al. 2022), but evidence indicates this can increase risk for problematic drinking and may exacerbate pain (Maleki et al. 2019). Much of the relevant empirical research on pain and these behavioural health challenges involves data collected over 3–6‐month (quarterly) time intervals (Saba et al. 2024, 2021) but symptoms can fluctuate daily (Schneider et al. 2012; Sullivan et al. 2020). Thus, data collected on a daily basis (i.e., daily diary data) could provide a more nuanced understanding of pain and its associations with stress and alcohol use (Asparouhov et al. 2018). Such data could clarify the day‐to‐day interplay between symptoms, as well as if there are additional factors that influence symptom associations (Davis et al. 2025).

One factor that might influence the day‐to‐day associations between pain and behavioural health symptoms including perceived stress and alcohol use among veterans is whether their pain becomes chronic and reaches clinically significant levels of severity. Here, it is important to clarify that individuals with chronic pain experience both trait‐level differences in typical pain levels (a veteran may have relatively high *chronic pain severity* on average compared to other veterans), but also state‐level fluctuations in daily pain (a veteran's own *pain intensity* can vary compared to their average pain level). Pain is assessed on a continuum—ranging from mild to moderate to severe (Williamson and Hoggart 2005)—and individuals with chronic pain of moderate or greater severity on average (hereafter referred to as moderate‐severe chronic pain) have been shown to experience greater difficulties coping with pain and worse behavioural health outcomes (Von Korff et al. 2000). Throughout this paper, we use *chronic pain severity* to refer to this trait‐like classification based on average pain levels, and *pain intensity* to refer to daily fluctuations in pain. Research has demonstrated that moderate‐severe chronic pain is prevalent among veterans and is associated with heightened behavioural health symptomology (Alschuler and Otis 2014; Nahin 2017).

While clinicians, including those who treat veterans, routinely assess pain severity to guide treatment decisions (Woo et al. 2015; Nassif et al. 2015; Pangarkar et al. 2019), we know surprisingly little about how behavioural health symptom associations may differ among veterans with moderate‐severe chronic pain compared to those with less severe pain. Allostatic load models, describing how chronic activation of the body's stress‐response systems leads to cumulative “wear and tear” on the body, may have implications for understanding associations between pain intensity, perceived stress, and alcohol use among those with moderate‐severe chronic pain (Mickle et al. 2023; McEWEN). From the perspective of these models, moderate‐severe chronic pain may reflect a state of physiological dysregulation where pain intensity and behavioural health symptoms like perceived stress and alcohol use may become more tightly coupled: when one symptom increases, dysregulated stress‐response systems may be less able to play a buffering role, potentially leading to stronger day‐to‐day associations between these symptoms (Mickle et al. 2023; McEWEN 1998). In parallel, the model of conservation of resources suggest that the persistent demands of moderate‐severe chronic pain may drain psychological coping resources, making it harder to manage on days when pain or behavioural health symptoms escalate, leading to cyclical increases in other symptoms (Hobfoll 1989). While direct tests of this model in chronic pain populations are limited, related research has shown that among individuals with chronic pain, poorer coping is associated with worse behavioural health symptoms (specifically, depression), consistent with a depletion effect (Kato et al. 2021). It remains unknown, however, whether veterans with moderate‐severe chronic pain experience distinct patterns of day‐to‐day associations between pain intensity, perceived stress, and alcohol use. Such knowledge could inform better targeted and timelier treatment for veterans with chronic pain.

Pain and perceived stress are closely linked in both the theoretical and empirical literature. Seminal theory on stress and coping suggests pain becomes stressful when it is perceived as threatening and too challenging to manage (Biggs et al. 2017), and empirical research has demonstrated heightened stress among those with chronic pain (Hallman and Lyskov 2012). The systems model of pain goes a step further and suggests a *bidirectional* association between pain intensity and perceived stress: as pain leads to increased stress, stress dysregulates key physiological systems, contributing to still greater pain (Chapman et al. 2008). One recent study of veterans reported evidence for bidirectional associations between perceived stress and pain intensity, but only among those who met criteria for an affective disorder and who, incidentally, had a relatively high average level of pain across the 18‐month study period (suggesting relatively severe chronic pain) (Saba et al. 2024). While this previous study used data collected over quarterly intervals, daily diary research with non‐veterans has also begun to demonstrate positive associations between pain and perceived stress measured at the daily level (e.g., among individuals who experience migraines (Vives‐Mestres et al. 2021)). Given that relatively severe chronic pain could substantially deplete coping resources and impair physiological regulation (Chapman et al. 2008; Crofford 2015; Hannibal and Bishop 2014), pain intensity and perceived stress may be intricately linked at the daily level, and this is *especially* worth exploring among high‐risk populations like veterans who often experience moderate‐severe chronic pain (Alschuler and Otis 2014; Nahin 2017).

Much has also been written on alcohol use among veterans with pain and multi‐morbid problems (Saba et al. 2021). The self‐medication model suggests that, while veterans may use alcohol to cope with pain/and or perceived stress, drinking can make these problems worse (Khantzian 1997). Prior quantitative and qualitative research with veterans has demonstrated positive associations between stress and alcohol use, in line with this model (Bouskill et al. 2022; Davis et al. 2022). A recent daily diary analysis revealed veterans' stress one day was associated with increased alcohol use the next day (Davis et al. 2025). Since excessive drinking may lead to stressful psychosocial consequences (e.g., work issues, relationship problems (Kendler et al. 2017)), there may also be a risk of a cycle (i.e., bidirectional association) between increased drinking and perceived stress. Associations between alcohol use and pain are similarly complex (E. L. Zale et al. 2015). While some research with non‐veterans has shown low‐risk alcohol consumption may reduce pain intensity (Scott et al. 2018), veterans with chronic pain have high rates of alcohol use disorder, suggesting drinking for pain relief may have deleterious consequences (Vowles et al. 2022). Some who self‐medicate may develop tolerance to alcohol (needing greater and greater amounts) and increased sensitivity to pain (De Aquino et al. 2024), potentially feeding a cycle of increased drinking and pain. These cyclical processes could be amplified among veterans with moderate‐severe chronic pain, who may be more likely to turn to alcohol to cope as their pain or stress gets worse or may suffer greater consequences from drinking. Daily diary data could elucidate these complex symptom dynamics and determine if they differ among veterans with moderate‐severe chronic pain compared to those with less severe pain (Davis et al. 2025).

Existing literature suggests pain intensity, perceived stress, and alcohol use are linked with one another in complex ways, likely at the daily level, underscoring the need for nuanced research. The present study is a secondary analysis of data on pain and behavioural health symptomatology among a sample of veterans engaged in a daily diary study. We aimed to explore the daily, multivariate associations between pain intensity, perceived stress, and alcohol use to provide a more nuanced understanding of how these multi‐morbid problems influence one another. More specifically, we aimed to explore whether associations differ between veterans with moderate‐severe chronic pain compared to those with less severe pain. We hypothesised that veterans with moderate‐severe chronic pain would demonstrate heightened, positive associations between daily study variables. Results can enhance understanding of the day‐to‐day experiences of symptomatology and coping among veterans with pain, to inform more targeted pain and stress management strategies.

## 2 Method

### 2.1 Participants and Procedures

Data are from a larger study of behavioural health symptom dynamics among US veterans, collected between January 2023 and February 2024 (Leightley et al. 2025). Participants (*n* = 74) were recruited via several channels including BuildClinical recruitment services and social media platforms, using previously developed measures to screen out those misrepresenting themselves as veterans (Pedersen et al. 2017). Inclusion criteria were: (a) U.S. veteran aged 18 or older; (b) separated or discharged from military service within the past 3 years; (c) previously served in the Air Force, Army, Marine Corps, or Navy; (d) not currently on active duty service or serving in the active reserves or national guard; (e) served in post‐9/11 conflicts (i.e., in Iraq/Afghanistan); (f) able to read English; (g) owns a smartphone released since 2012 with Internet access and have interest in using apps on that phone; (h) not receiving treatment for cannabis, alcohol, or other drug use or PTSD at the VA or other health care provider; (i) use of cannabis in the prior month, (j) experienced a traumatic event during military service, and (k) had a Primary Care PTSD screen score of 1 or more, representing at least one PTSD symptom cluster (Prins et al. 2004). Study method and results are reported following the Strengthening the Reporting of Observational Studies in Epidemiology (STROBE) Statement for observational studies (Elm et al. 2007).

Data on participants' symptomology were collected via the smartphone application MAVERICK, which was developed by digital mental health investigators at King's College London. Data were collected at baseline and twice daily for 3 months: during the morning (regarding symptoms experienced during the prior evening/night, between 6p.m. and 6a.m.) and during the evening (regarding symptoms experienced during that day, between 6a.m. and 6p.m.). Survey reminders were sent via smartphone push notification and text messaging. Participants were paid $20 for completing the baseline survey and $1 per day for both daily surveys. Participants provided informed consent prior to parent study enrolment and data collection.

See Table 1 for participant demographics and clinical characteristics at baseline. Study participants were 81% male and were age 33.5 on average (SD = 8.2). The racial make‐up of participants was 61% White, 10% Black, and the remaining (29%) mixed race or some other race, and 36% of participants were of Hispanic/Latinx ethnicity. On average, participants had experienced 2.6 deployments (SD = 2.9). At baseline, participants reported using alcohol an average of 8.6 out of the past 30 days (SD = 8.25) and had 3.7 drinks (SD = 3.4) on days they consumed alcohol. The mean pain intensity score on the PROMIS Pain Intensity (short form) measure was 7.53 (SD = 2.81) representing mild‐moderate pain on average over the past 7 days (Stone et al. 2016). Individuals provided 58.2 days (SD = 31.47) of data on average (out of a total of 87 possible days), for a total of 4307 days of data across the sample.

**TABLE 1:** Participant characteristics.

	Mean or *N* (SD or %)
Age, years	33.52 (8.17)
Sex	
Male	58 (80.56%)
Female	14 (19.44%)
Ethnicity	
Hispanic/latino	26 (36.11%)
Not hispanic/latino	46 (63.89%)
Race	
White	44 (61.11%)
Black	7 (9.72%)
Asian	2 (2.78%)
Native american	1 (1.39%)
Mixed	10 (13.89%)
Other	8 (11.11%)
Employment status	
Working full‐time	24 (33.33%)
Working part‐time	19 (26.39%)
Unemployed, looking for work	9 (12.5%)
Unemployed, not looking for work	8 (11.11%)
Retired	12 (16.66%)
Number of deployments	2.55 (2.93)
Baseline clinical characteristics	
Pain, average over past 7 days	7.58 (2.81)
Frequency of alcohol use, days per past 30 days	8.59 (8.25)
Quantity of alcohol use, average per day	3.69 (3.38)
Daily clinical characteristics, average	
Pain intensity	3.12 (2.28)
Alcohol use quantity	0.61 (0.90)
Perceived stress	1.94 (1.07)

*Note:* Frequency tables add up to 72 (rather than 74) as two participants did not complete the baseline survey. Age in years, biological sex at birth, race, ethnicity, and employment status are self‐reported. At baseline, pain is measured with the PROMIS pain intensity (short form), capturing average pain over the past 7 days (range: 3–15). Frequency and quantity of alcohol use were each measured with a single item asking how often in the past 30 days the participant had a drink containing alcohol, and how many drinks of alcohol they had on days they drank, respectively. Daily clinical characteristics were measured each day during the study period with single items for pain intensity (numerical rating scale, range: 0–10), alcohol use quantity (number of drinks, range: 0–30), and perceived stress (severity of stress, range: 0–5); provided are sample mean and SD of person‐level means of their daily clinical characteristics across the study period.

### 2.2 Measures

#### 2.2.1 Baseline Characteristics

Participants' self‐reported sex at birth, race and ethnicity, and military characteristics (such as branch, number of combat deployments, time since last deployment) were collected at baseline. Alcohol use was reported as the total number of days used out of the prior 30 days participants had a drink containing alcohol, and their average number of drinks per day during that period. Perceived stress over the past month was assessed with the 10‐item Perceived Stress Scale (PSS (Lee 2012)). Pain intensity over the past 7 days was assessed using the three‐item PROMIS Pain Intensity (short form) measure (Stone et al. 2016).

#### 2.2.2 Pain Intensity (Daily) and Chronic Pain Severity

The single‐item numerical rating scale of pain intensity was used to measure daily pain intensity during each of two daily surveys (Woo et al. 2015). Participants were asked to rate their pain during the prior 12‐h period, using a simple slider, from 0 (no pain) to 10 (worst pain ever). The single‐item numerical rating scale has been validated widely in clinical and research contexts (Williamson and Hoggart 2005).

Responses were averaged for a composite score of daily pain intensity. To determine whether participants had moderate‐severe chronic pain on average throughout the 3‐month study period, daily pain scores were averaged over study period, and those with an average score of 4 or above were considered to have moderate‐severe chronic pain, in line with the definition of chronic pain (Treede et al. 2015) and established cut points for intensity (Woo et al. 2015). This approach captures average pain intensity across repeated daily reports over the 3‐month observation period, consistent with established definitions of chronic pain as lasting 3 months or more (Treede et al. 2015).

#### 2.2.3 Perceived Stress (Daily)

During each daily survey, participants were asked to respond to a single item indicating how stressed they felt during the prior 12‐h period using a simple slider (range from 0 “not at all” to 5 “extremely”). Responses were averaged for a composite score of daily perceived stress.

#### 2.2.4 Alcohol Use (Daily)

During each daily survey, participants responded to a single item enquiring about the number of US standard alcoholic drinks they had during the prior 12‐h (range from 0 to 30 drinks). Responses were averaged for a composite score of daily alcohol use.

### 2.3 Analytic Plan

The present study utilises dynamic structural equation modelling (DSEM), which merges time series analysis, multilevel modelling, structural equation modelling, and time‐varying effects modelling (Asparouhov et al. 2018). DSEM was designed for intensive longitudinal data where many observations are nested within a single person, and tests between‐ and within‐person associations in a single model. Unlike traditional multilevel models, DSEM allows for structural associations between multiple outcomes (allowing for bidirectional effects). Analyses were conducted in M*plus* version 8.6 (van derLinden 2018).

Prior to analyses, data was aggregated at the daily level by averaging each day's two scores on a variable if an individual responded to both surveys, or by using the single available score if an individual responded to one survey. Days with no responses were marked as missing. DSEM treats remaining missing data similarly to random effects, where an individual's missing values on a given day are estimated based on the neighbouring days and the individual's autoregressive parameter.

As a preliminary modelling step, stationarity of pain intensity, perceived stress, and alcohol use was assessed as DSEM assumes stationarity (i.e., that the means, variance, and autocorrelations of study variables do not systematically change over time (Falkenström et al. 2017)). As time trends were detected for each study variable, to de‐trend the data a residualized DSEM (RDSEM) model was employed where time (number of days since the start of the study) was included as a covariate on pain intensity, perceived stress, and alcohol use, and associations between residuals were estimated.

To model the daily, dynamic associations between pain intensity, perceived stress, and alcohol use, and explore whether parameters differed between those with moderate‐severe chronic pain versus those with lower levels of pain, we fit a trivariate, multi‐group RDSEM. This model includes within‐person, autoregressive parameters (the same construct predicting itself on the subsequent day) and cross‐lagged parameters (one construct predicting another construct on the subsequent day) for all three constructs. Models also included within‐person correlations (correlations between an individual's scores on each construct on the same day) and between‐person correlations (correlations between an individual's average scores on each construct, compared with the rest of the sample). The model was fit as a multi‐group model where moderate‐severe chronic pain (yes/no) was a grouping variable, using methodology outlined by the developers of DSEM (Asparouhov and Muthén 2010). Participants' self‐reported age, biological sex, race/ethnicity, and number of deployments were included as covariates on the between‐person parameters for pain intensity, perceived stress, and alcohol use.

Model convergence was checked using the Potential Scale Reduction (PSR) value. The PSR is calculated by comparing the variance within Bayesian Markov Chain Monte Carlo chains to the variance between chains. A PSR close to 1.00 indicates that the chains are converging well, suggesting parameter estimates are reliable, thus M*plus* provides model output after the PSR drops close below 1.10. In line with recommendations, after output was provided the model was rerun with double the number of iterations to ensure that the PSR value did not increase.

## 3 Results

Thirty‐six percent of participants (*n* = 27) met criteria for moderate‐severe chronic pain, and they had a mean daily pain score of 5.64 throughout the study period on average, representing moderate chronic pain on average per established cut points (Woo et al. 2015); 64% of participants (*n* = 47) had less severe pain, and they had a mean daily pain score of 1.68 throughout the study period on average, representing mild pain on average. Baseline characteristics were compared between those with moderate‐severe chronic pain versus those with less severe pain using *t*‐tests and chi‐square tests. The groups did not differ on demographic or baseline clinical characteristics except past 7‐day pain intensity.

**FIGURE 1:**
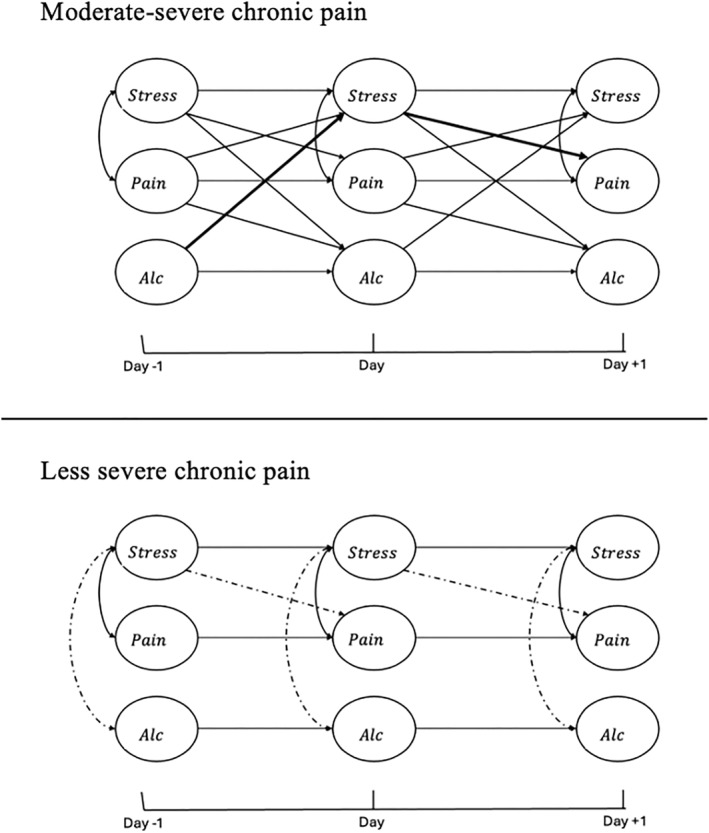
Diagram of significant multigroup RDSEM results with post‐hoc, indirect effect. alc = alcohol use; pain = pain intensity; Stress = perceived stress. All within‐person day‐to‐day autoregressions, day‐to‐day cross‐lags, and same day covariances were estimated. Only significant paths are included in the diagram for readability. Solid lines indicate positive associations, and dashed lines indicate negative associations. Bold lines indicate the significant, positive indirect path tested in post‐hoc analyses (alc → stress → pain among those with moderate‐severe chronic pain).

The multigroup RDSEM model converged after 10,000 iterations with the PSR dropping below 1.10, and the PSR dropped below 1.03 after the iterations were doubled to 20,000. Table 2 provides model results with unstandardised parameter estimates and standard errors for the final model, and Figure 1 provides a simplified visual diagram of significant associations. In the text below, unstandardised posterior median estimates (b) and 95% credible intervals (CI) are reported for lagged effects, and correlations are represented by *φ*_*standardized*_.

**TABLE 2:** Final multigroup RDSEM estimates for associations between daily pain intensity, perceived stress, and alcohol use, by chronic pain severity.

Estimate	Moderate‐severe chronic pain		Less severe pain	
	Estimate	95% CI	Estimate	95% CI
Within‐person day cross‐lags				
Pain intensity_*t‐1*_ → perceived stress_*t*_	**0.06**	**(0.02, 0.10)**	−0.01	(−0.04, 0.02)
Perceived stress_*t‐1*_ → pain intensity_*t*_	**0.13**	**(0.06, 0.20)**	**−0.05**	**(−0.09, −0.002)**
Pain intensity_*t‐1*_ → alcohol use_*t*_	**0.04**	**(0.01, 0.08)**	0.01	(−0.03, 0.05)
Alcohol use_*t‐1*_ → pain intensity_*t*_	0.04	(−0.04, 0.12)	−0.01	(−0.05, 0.02)
Perceived stress_*t‐1*_ → alcohol use_*t*_	**0.05**	**(0.01, 0.10)**	−0.01	(−0.06, 0.04)
Alcohol use_*t‐1*_ → perceived stress_*t*_	**0.07**	**(0.01, 0.13)**	0.02	(−0.01, 0.04)
Within‐person day autoregressions				
Pain intensity_*t‐1*_ → pain intensity_*t*_	**0.32**	**(0.27, 0.38)**	**0.55**	**(0.51, 0.58)**
Perceived stress_*t‐1*_ → perceived stress_*t*_	**0.28**	**(0.23, 0.33)**	**0.40**	**(0.36, 0.44)**
Alcohol use_*t‐1*_ → alcohol use_*t*_	**0.13**	**(0.08, 0.19)**	**0.11**	**(0.07, 0.15)**
Within‐person day (co)variances				
Pain intensity variance	**1.91**	**(1.77, 2.06)**	**0.79**	**(0.75, 0.84)**
Perceived stress variance	**1.08**	**(1.00, 1.16)**	**0.56**	**(0.53, 0.59)**
Alcohol use variance	**0.90**	**(0.84, 0.97)**	**1.12**	**(1.06, 1.19)**
Pain intensity with perceived stress	**0.26**	**(0.19, 0.35)**	**0.13**	**(0.11, 0.16)**
Pain intensity with alcohol use	0.03	(−0.04, 0.10)	−0.02	(−0.06, 0.02)
Perceived stress with alcohol use	0.02	(−0.03, 0.07)	**−0.05**	**(−0.08, −0.02)**
Within‐person stationarity				
Day number → pain intensity_*t*_	**0.012**	**(0.01, 0.02)**	0.001	(−0.004, 0.01)
Day number → perceived stress_*t*_	0.004	(−0.001, 0.01)	**−0.004**	**(−0.01, −0.001)**
Day number → alcohol use_*t*_	0.003	(−0.001, 0.01)	**−0.01**	**(−0.01, −0.002)**
Between‐person intercepts and variances				
Pain intensity intercept	**4.46**	**(0.87, 7.79)**	−0.04	(−1.89, 1.83)
Perceived stress intercept	**3.17**	**(1.85, 5.09)**	0.87	(−0.82, 2.56)
Alcohol use intercept	0.80	(−1.74, 3.22)	−0.26	(−1.57, 1.07)
Pain intensity variance	**2.78**	**(1.42, 6.36)**	**1.21**	**(0.76, 2.09)**
Perceived stress variance	**1.12**	**(0.58, 2.32)**	**1.12**	**(0.72, 1.82)**
Alcohol use variance	**1.62**	**(0.83, 3.48)**	**0.61**	**(0.36, 1.08)**
Fit statistics				
Free parameters	94			
DIC	87033.02			
Number of iterations	20,000			
PSR value	1.029			

*Note:* Pain intensity was measured using the numerical rating scale from 0 to 10, with higher scores indicating more intense pain; Perceived stress was measured using a slider scale from 1–5, with higher scores indicating higher levels of perceived stress; Alcohol use was measured as the number of drinks a participant reported on each daily survey. Unstandardised posterior median estimates and 95% credible intervals provided from final multigroup RDSEM model capturing associations between residualized pain intensity, perceived stress, and pain at the daily level, after controlling for day number to account for time trends due to non‐stationarity (see “Within‐person stationarity” estimates for time trends depicting the associations between day number and study constructs). Results are provided for those with moderate‐severe chronic pain and less severe pain in one model; moderate‐severe chronic pain is defined as having an average pain level of 4 or above throughout the 3‐month study period. Estimates for other control variables (age, minority race/ethnicity, female sex, number of deployments) on between‐person mean parameters not shown for readability. Subscripts (*t*) identify time of measurement. Bold = significant, 95% credible interval does not include 0.

Abbreviations: DIC = deviance information criterion; PSR = potential scale reduction.

### 3.1 Daily Symptom Dynamics for Veterans With Moderate‐Severe Chronic Pain

Among veterans with moderate‐severe chronic pain, there were several significant *lagged (day‐to‐day)* associations between study variables. Pain intensity and perceived stress were bidirectionally, positively associated: on days veterans reported higher perceived stress than their own average, they reported higher pain intensity the following day (*b* = 0.13, 95% CI = [0.06, 0.20]); and, on days they reported higher pain intensity than their own average, they reported higher perceived stress the following day (*b* = 0.06, 95% CI = [0.02, 0.10]). There was a positive association between pain intensity and alcohol use, such that on days they reported higher pain intensity than their own average, they reported consuming a greater number of drinks the following day (*b* = 0.04, 95% CI = [0.01, 0.08]). Finally, perceived stress and alcohol use were bidirectionally, positively associated: on days veterans reported higher perceived stress than their own average, they reported consuming a greater number of drinks the following day (*b* = 0.05, 95% CI = [0.01, 0.10]); and, on days they reported consuming a greater number of drinks than their own average, they reported higher perceived stress the following day (*b* = 0.07, 95% CI = [0.01, 0.13]). In terms of *contemporaneous (same‐day)* associations, there was a significant positive correlation between pain intensity and perceived stress, such that on days veterans reported higher pain intensity than their own average they also reported higher perceived stress than their own average (*φ*_*standardized*_ = 0.19).

### 3.2 Daily Symptom Dynamics for Veterans With Less Severe Pain

Among veterans with less severe pain throughout the study period, there was only one significant lagged association. Perceived stress was negatively associated with pain intensity the following day: on days veterans reported higher perceived stress than their own average, they reported lower pain intensity the following day (*b* = −0.05, 95% CI = [−0.09, −0.002]). In terms of same‐day associations, there was a significant positive correlation between pain intensity and perceived stress, such that on days veterans reported higher pain intensity than their own average they also reported higher perceived stress than their own average (*φ*_*standardized*_ = 0.20). There was also a significant negative correlation between alcohol use and perceived stress, such that on days veterans reported higher alcohol use than their own average they reported lower perceived stress than their own average (*φ*_*standardized*_ = −0.07).

### 3.3 Post Hoc Analysis: Indirect Effect Linking Alcohol Use and Pain Intensity Among Veterans With Moderate‐Severe Chronic Pain

In the final model, among the moderate‐severe chronic pain group, all but one lagged association between pairs of variables were significant and positive: the lagged association between alcohol use one day and pain intensity the following day. However, when examining our final model, it appeared that perceived stress may be acting as a mechanism of change linking alcohol use and pain intensity over a 3‐day period (see Figure 1). To explore if stress is acting as a mechanism of change, we estimated an indirect effect. There was indeed a positive indirect effect linking alcohol use and pain intensity via perceived stress (*b* = 0.01, 95% CI = [0.001, 0.02]). That is, on days veterans with moderate‐severe chronic pain reported higher alcohol use than their own average, they are expected to report higher perceived stress the next day; and subsequently, as they had higher perceived stress then their own average on the second day, they are expected to experience higher pain than their own average on the third day.

## 4 Discussion

Prior research has demonstrated links between pain and behavioural health symptoms, including alcohol use and perceived stress, among veterans (Saba et al. 2021; Davis et al. 2025, 2022). We extend this literature to enhance understanding of these symptoms among veterans, exploring multivariate associations at the daily level to better match the real‐world complexity of symptomology (Asparouhov et al. 2018). While prior literature makes clear that veterans with relatively severe chronic pain also tend to have more severe behavioural health problems on average (Otis et al. 2010; Saba et al. 2021), we demonstrate that those with moderate‐severe chronic pain also likely experience more challenging day‐to‐day symptom *dynamics*. Specifically, there were significant, positive associations between nearly all pairs of study constructs (pain intensity, perceived stress, and alcohol use) among veterans in our sample with moderate‐severe chronic pain—in line with our hypothesis—and this was not the case among veterans with less severe pain. These results suggest that as pain becomes chronic and reaches greater clinical severity, a veteran's day‐to‐day changes in pain intensity and behavioural health symptoms may become more entangled with one another. This pattern is consistent with theories on allostatic load and resource conservation, whereby veterans with moderate‐severe chronic pain experience cumulative physiological “wear and tear” and reduced coping resources due to repeated activation of stress‐response systems over time, perhaps making it more challenging to regulate increases in symptomology (Mickle et al. 2023; McEWEN 1998; Hobfoll 1989).

Our results demonstrate close links between pain intensity and perceived stress–especially among veterans with moderate‐severe chronic pain. For all veterans in the sample, on days in which pain was high, perceived stress was also high. However, for those with moderate‐severe chronic pain, there were also bidirectional *lagged* associations, where greater pain intensity predicted subsequent perceived stress, and stress predicted subsequent pain. This result is in line with the systems model of pain, which suggests pain and stress amplify one another and are cyclically linked (where pain makes stress worse, and vice versa (Chapman et al. 2008)). One prior study also reported a bidirectional lagged association between pain intensity and perceived stress, measured at different time interval (3‐month), among veterans with moderate‐severe depressive disorder (Saba et al. 2024). We extend this literature by showing bidirectional associations between pain and stress can be detected at different time intervals, and notably, among another group of veterans with clinically significant symptomology (those with moderate‐severe chronic pain). Taken together, both study's findings suggest that relatively severe clinical problems–either physical or behavioural health–might influence stress reactivity and how one copes with stress and pain, to alter daily pain and stress dynamics (Chapman et al. 2008). In fact, among those in our sample with lower average levels of pain, experiencing perceived stress that was higher than one's own average predicted *reduced* pain intensity the following day, perhaps because these veterans were able to mobilise effective stress management behaviours, and the effects spilt over to the next day and reduced pain.

There was also ample evidence for the self‐medication model at the daily level among veterans with moderate‐severe chronic pain. For these veterans, heightened pain intensity or perceived stress one day predicted increased alcohol use the next day. However, heightened alcohol use in turn also predicted increased perceived stress one day later, which could itself lead to increased pain intensity on the third day (demonstrated by the significant, indirect association between alcohol use and pain intensity via perceived stress). This is in line with prior work indicating individuals use substances to cope with physical or psychological distress, but then substance use can ultimately make distress worse (Khantzian 1997). While other studies have shown links between pain intensity and both perceived stress and alcohol use, measured in 3–6‐month intervals, among veterans (Vowles et al. 2022; Davis et al. 2022), ours is the first to explore daily symptom dynamics involving all three constructs, and we extend knowledge by demonstrating distinct symptom dynamics among veterans with moderate‐severe chronic pain. That is, our results highlight a possible “catch 22” scenario for these veterans where they may be more likely to use alcohol to cope with increases in pain intensity or perceived stress, but they also may be more likely to suffer negative consequences–including *more* pain and stress–from drinking. Among veterans with less severe pain on average, on the other hand, on days they drank more alcohol than their own average, they also had lower perceived stress than their own average–suggesting alcohol use may on some level “work” for stress management for those with less severe pain.

Our results should be interpreted in light of methodological decisions and their limitations. Despite the fact that DSEM is a sophisticated modelling strategy that can estimate within‐person associations–effectively controlling for any fixed, between‐person differences among participants–one should still be cautious about drawing causal conclusions from this observational study. It is likely that changes in measured symptoms do not entirely explain changes in other symptoms among those with moderate‐severe chronic pain, as additional factors that vary day‐to‐day such as social support, physical activity levels, and sleep might also influence pain, perceived stress, and alcohol use concurrently. And while studying these symptom associations at the daily level is important and carries clear intervention implications given that pain and behavioural health are known to fluctuate daily, it is possible that associations might differ at intervals that are shorter (e.g., hourly or moment‐to‐moment) or longer (weekly or monthly).

Relatedly, the single item for pain intensity likely does not fully capture the daily experience of pain, given the multidimensionality of pain and that patients with some pain conditions report single item measures may not capture all changes due to symptom fluctuations (Hawker et al. 2011). Measures of pain interference, or the extent to which pain interferes with day‐to‐day functioning, provide especially useful clinical information (Jensen et al. 2017), and future work should explore symptom associations involving daily pain interference. It is also possible that we were unable to capture some symptom associations in the less severe pain group due to a lower ceiling for pain intensity, though we note that there was significant daily variance in pain intensity in this group and one significant lagged association that ran counter to the moderate‐severe chronic pain group (a *negative* association between perceived stress and pain intensity)—highlighting a clear pattern in our results.

Finally, our sample comprised predominantly White male veterans with baseline behavioural health symptoms, namely some PTSD symptomology and a recent history of cannabis use, at baseline. Our sample is similar in demographic characteristics to the population of post‐9/11 U.S. veterans, a substantial proportion of which have PTSD symptoms and use cannabis (Meffert et al. 2019; Hill et al. 2021). Still, generalisability to more diverse subpopulations of veterans or those with different clinical characteristics may be limited.

Despite these limitations, our results illustrate clear differences in daily associations between pain intensity, perceived stress, and alcohol use among veterans with moderate‐severe chronic pain compared to those with less severe pain levels, and they carry important clinical implications. Among those with moderate‐severe chronic pain, an increase in one symptom (whether it be pain intensity, perceived stress, or alcohol use) appears to precipitate increases in virtually all other symptoms, suggesting that this group is at risk of a cascade of worsening of symptomatology over relatively short (day‐to‐day) time intervals. This suggests a need to prioritise prevention to the extent possible, that is, for veterans with moderate‐severe chronic pain to be aware of what makes pain and behavioural health worse and to utilise strategies that minimise risk of worsening symptomology. These veterans may especially benefit from better learning to manage stress and pain, and clinicians should explicitly discuss alternatives to alcohol for coping–particularly given that alcohol might make their symptomology worse. Treatments like mindfulness‐based interventions and cognitive behavioural therapy could target transdiagnostic processes implicated in pain, perceived stress, and alcohol use (Greeson et al. 2014; E. Zale 2021), but there is a need to more broadly educate veterans with chronic pain and providers on the usefulness of these interventions and to ensure veterans can deploy these strategies in their day‐to‐day lives as needed to address acute symptomology (Goldsmith et al. 2023). All in all, results are clearly in line with the biopsychosocial model, emphasising the need for whole‐person, holistic care and responsive self‐management strategies that enable veterans with chronic pain to monitor and address day‐to‐day fluctuations in symptoms.

## Funding

This research was supported by grants from the National Institutes of Health (F31DA058105 to SKS and R21DA051802 to JPD).

## Ethics Statement

All procedures performed in studies involving human participants were in accordance with the ethical standards of the institutional research committee and with the 1964 Helsinki declaration and its later amendments or comparable ethical standards.

## Consent

This manuscript reports a secondary analysis of de‐identified data; all participants provided informed consent prior to parent study enrolment and data collection.

## Conflicts of Interest

The authors declare no conflicts of interest.

## Permission to Reproduce Material From Other Sources

The authors declare no conflicts of interest.

## Data Availability

De‐identified data from this study are not available in a public archive. De‐identified data from this study may be made available (as allowable according to institutional IRB standards) by emailing the corresponding author.
